# Eustachian Tube Foreign Body with Endoscopic-Assisted Surgical Removal

**DOI:** 10.1155/2019/5236429

**Published:** 2019-12-27

**Authors:** Phillip R. Purnell, Adam Bender-Heine, Habib Zalzal, Abdul R. Tarabishy, Adam Cassis

**Affiliations:** ^1^West Virginia University, Department of Otolaryngology-Head and Neck Surgery, Morgantown, WV 26505, USA; ^2^West Virginia University, Department of Radiology, Morgantown, WV 26505, USA

## Abstract

**Objectives:**

Foreign bodies of the external and middle ear are not uncommon; however, foreign bodies in the eustachian tube are rare. Here we describe the presentation, imaging, and endoscopic-assisted surgical management of a case of eustachian tube foreign body.

**Methods:**

A 34-year-old male was seen for evaluation of foreign body of the left eustachian tube while working with metal at a machine shop. Imaging and surgical management are highlighted and review of available literature regarding foreign bodies of the eustachian tube is presented.

**Results:**

A CT scan revealed a foreign body present approximately 1 cm into the bony eustachian tube. The patient underwent middle ear exploration which required endoscopic assistance to adequately visualize the foreign body. The foreign body was unable to be removed and required the creation of a bony tunnel lateral to the eustachian tube for visualization and access to the foreign body.

**Conclusions:**

This report presents a rare case of eustachian tube foreign body. Use of the endoscope during the surgical removal greatly enhanced the ease and safety of removal. This report also highlights the importance of ear protection with any machining and welding work.

## 1. Introduction

Foreign bodies of the external ear canal and middle ear are not uncommon [1]. However, foreign bodies found in the eustachian tube are much rarer. In the past century, foreign bodies of the eustachian tube were more common when eustachian tube dilation (bugeonage) was still being practiced with metal dilation and electrolytic therapy. Eustachian tube dilation was first described in the 1700s by several surgeons for many otologic ailments [2]. This practice declined greatly in the early 20^th^ century although otologists continued to identify broken dilator tips as eustachian tube foreign bodies for many years [3, 4]. Welding accidents involving the outer ear and tympanic membrane (TM) have been described and treated by many otologists [5]. Here we describe a rare presentation of foreign object in the eustachian tube afterwhile working with metal at a machine shop. We believe that this is the second case reported in the literature of metal foreign object in the eustachian tube with welding and machine work [6]. Our surgical approach, imaging, and the use of endoscopic assistance are highlighted.

## 2. Case Presentation

**Figure 1:**
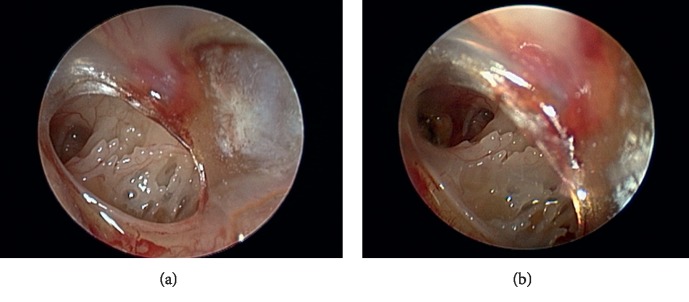
Left-sided anterior-inferior tympanic membrane perforation with area of trauma at the entrance to the bony eustachian tube. (a) View with 0-degree endoscope. (b) View with 30-degree endoscope.

**Figure 2:**
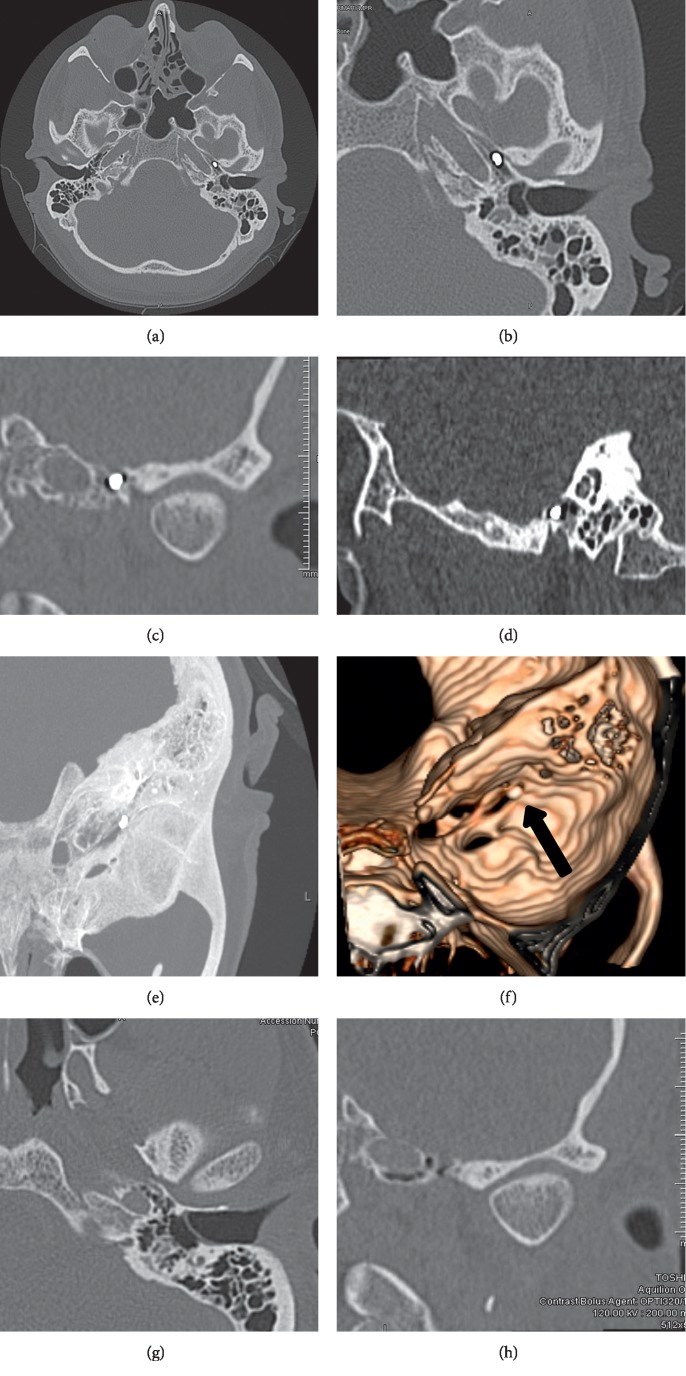
Coronal, axial, and sagittal CTs demonstrating radiopaque foreign body at the level of the bony eustachian tube. (a) Axial CT demonstrating foreign body in the left eustachian tube with normal appearance of the right eustachian tube. (b) Magnified axial CT also demonstrating the proximity to the carotid artery. (c) Coronal CT with object in the left bony eustachian tube. (d) Sagittal CT. (e) Oblique MIP in the same plane as the 3D reconstruction. (f) 3D reconstruction with arrow to the foreign body. (g) Postoperative axial CT. (h) Postoperative coronal CT.

A 34-year-old male presented to the emergency department with a penetrating injury of a metallic shard to the left ear while working as a machinist. He was not wearing hearing protection at the time. He was experiencing acute left-sided otalgia, decreased hearing on the left, change in taste, and mild imbalance. The patient had no previous ear surgery and an unremarkable past medical history apart from bilateral loud noise exposure while in the military. Physical exam showed a 40% anterior inferior quadrant perforation of the left TM with some swelling around the eustachian tube (Figure 1). Otolaryngology was consulted and a computed tomography (CT) scan of the temporal bones was obtained. The CT scan showed a 3 mm metallic object lodged in the left eustachian tube (Figures 2(a)–2(d)). The maximal intensity projection (MIP) and the 3D reconstruction are shown in Figures 2(e) and 2(f). The patient was started on a steroid and antibiotic ear drops. The patient was seen in the Otolaryngology clinic later that week for an audiogram and found to have a significant left-sided mixed hearing loss with a large air-bone gap (Supplemental [Supplementary-material supplementary-material-1]). Surgical removal was discussed, and the patient was consented for a left middle ear and eustachian tube exploration with a plan for lateral graft tympanoplasty and endoscopic-assisted removal of the foreign body.

**Figure 3:**
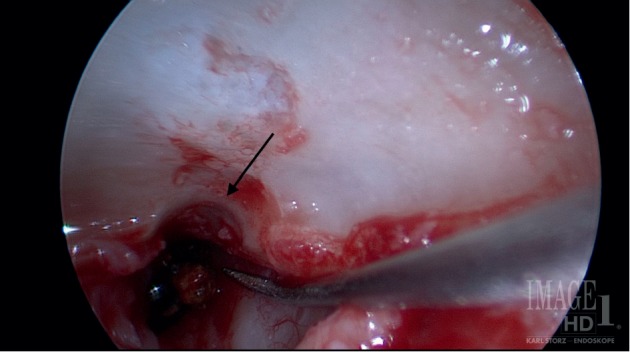
Endoscopic view of the foreign body in the eustachian tube. Please see the video online for further surgical views.

At the time of surgery ([Supplementary-material supplementary-material-1]), a 0-degree endoscope was used to visualize the left eustachian tube orifice through the ear canal. A curved olive-tipped suction was introduced into the left eustachian tube orifice and irrigated to move the metallic foreign body into the left middle ear. A standard left transcanal vascular strip was incised and back elevated followed by a postauricular approach to the left EAC. The left EAC skin was elevated and the remaining TM was elevated from the ossicles and annulus proper. A canaloplasty was performed, and the chorda tympani and ossicular chain were intact. Near the eustachian tube, the middle ear mucosa was edematous but healthy appearing. Initially, the microscope was utilized to try to remove the foreign body, but the metal slag was unable to be mobilized or removed using this approach. Both 0- and 30-degree endoscopes were then utilized to improve visualization, but access was still limited due to inability to manipulate the foreign body. To improve access, a bony tunnel was drilled just lateral to the eustachian tube orifice within the tympanic annular groove (Figure 3). This bony notch facilitated better access and instrumentation, particularly with a 0-degree endoscope. Through the notch, a Rosen needle was used to unseat and remove the foreign body from the eustachian tube. The middle ear was then packed with saline-soaked Gelfoam prior to placing a previously harvested true temporalis fascia graft in overlay fashion. The skin was returned anteriorly to its usual position within the ear canal. Postoperative CT scans were obtained several months later, showing removal of the object (Figures 2(g) and 2(h)). Audiograms demonstrating Type A tympanogram with continued sensorineural hearing loss are shown in Supplemental [Supplementary-material supplementary-material-1].

## 3. Discussion

There have been very few accounts of foreign body placement within the eustachian tube since the end of eustachian tube dilation and catheterization procedures in the 20^th^ century. Prior to this time, the tip of a dilator was frequently broken off in the eustachian tube during catheterization [2, 4]. Since that time, descriptions of eustachian tube foreign bodies have been limited to unfortunate trauma after assault [7], an acupuncture bead [8], and a similar metal slag from welding [6].

Most otologic injuries in welding are secondary to burn injuries along the pinna and TM perforations causing scarring and devascularization [9]. In most patients, rarely does a foreign body retain itself within the middle ear nor within the eustachian tube, but due to the devascularization of the tympanic membrane, these injuries are difficult to repair. As a result, a lateral graft/overlay tympanoplasty was necessary for our patient after debriding nonviable tissue surrounding the preexisting traumatic perforation. In working up this patient, a metallic slag could be confirmed on the CT scan due to the high intensity of metal with this type of imaging. It is important to note that ordering magnetic resonance imaging (MRI) in this patient may have resulted in further damage to the patient due to magnetic field interaction with metal, and it is recommended not to order an MRI if there is concern for metallic-based injury. Magnetic interaction with metal will not only result in thermal injury causing adhesion and obliteration of the eustachian tube but could also result in proximity damage to the adjacently positioned internal carotid artery. Inner ear (deafness and vestibular weakness) and facial nerve damage secondary to thermal injury have also been described in patients suffering from postwelding spark or metallic slag introduction to the middle ear [10].

Our case report describes the presentation of a metallic foreign body that lodged itself in the bony eustachian tube after passing through the TM. Removal required drilling lateral to the eustachian tube in order to gain access for removal. The use of the endoscope was key to successful removal and to avoid any damage to the adjacent carotid artery. The edema and scarring caused by the hot welding slag increased the difficulty of removal. Metal slag TM perforation is unlikely to heal on its own, and lateral graft tympanoplasty was completed at the same time. This case is also a reminder of the importance of ear protection to avoid possible transtympanic trauma with welding or machine work.

## Supplementary Materials

Supplementary Materials Imaging of the endoscopic removal of the foreign body from the eustachian tube complete with CT imaging and postoperative photos. Supplemental Figure 1: (A) preoperative audiogram; (B) preoperative tympanogram; (C) postoperative audiogram; (D) postoperative tympanogram (left-blue, right-red).
