# Supplementary figures and images for: Eustachian Tube Foreign Body with Endoscopic-Assisted Surgical Removal
Source: Case Rep Otolaryngol. 2019 Dec 27;2019:5236429. doi: 10.1155/2019/5236429 (PMC6949665; doi:10.1155/2019/5236429)

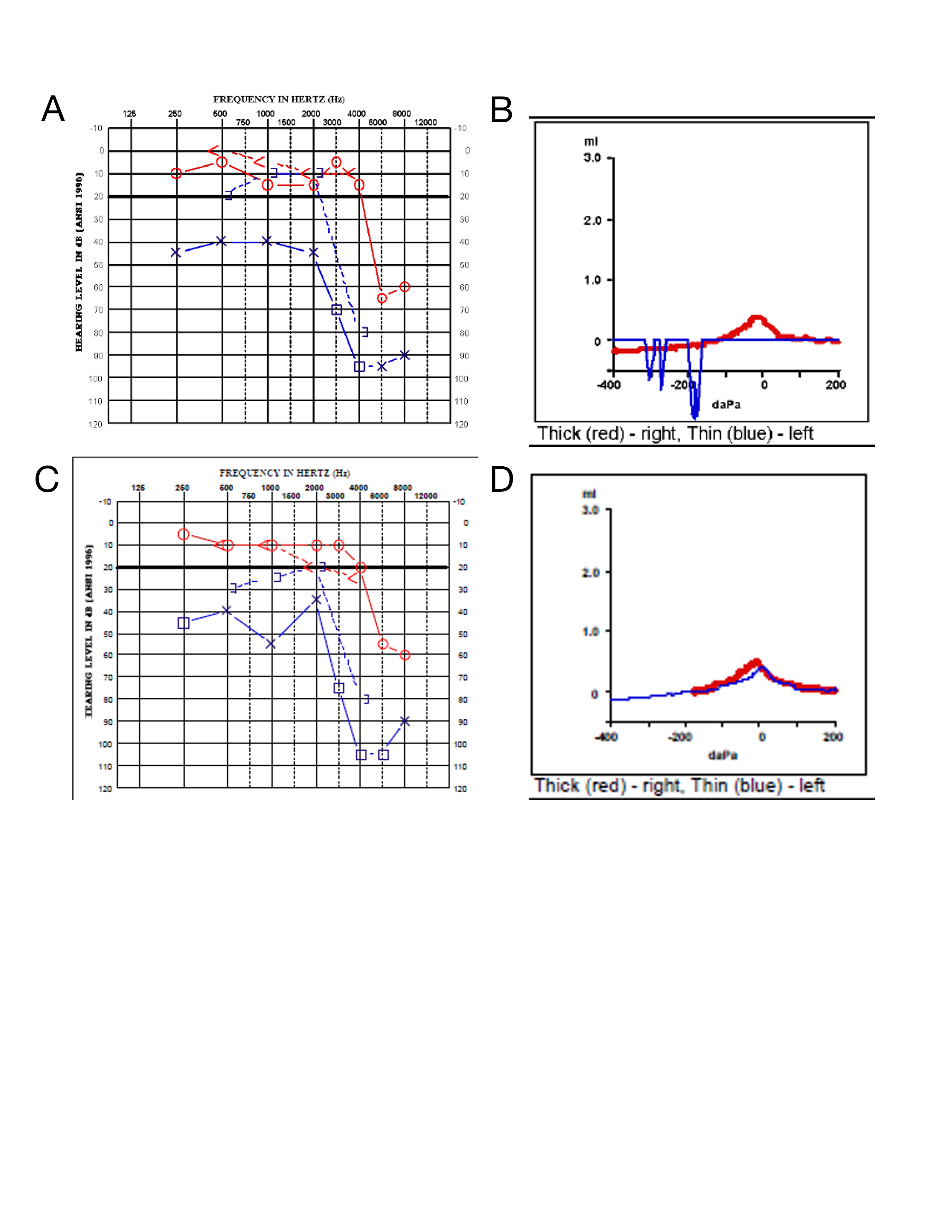

Supplement: Supplementary Materials — Imaging of the endoscopic removal of the foreign body from the eustachian tube complete with CT imaging and postoperative photos. Supplemental Figure 1: (A) preoperative audiogram; (B) preoperative tympanogram; (C) postoperative audiogram; (D) postoperative tympanogram (left-blue, right-red). [file 5236429.f1.zip › 5236429.f1/Supplemental Figure 1 _CRIOT_2956648.jpg]
